# AFM Force Relaxation Curve Reveals That the Decrease of Membrane Tension Is the Essential Reason for the Softening of Cancer Cells

**DOI:** 10.3389/fcell.2021.663021

**Published:** 2021-05-12

**Authors:** Keli Ren, Jingwei Gao, Dong Han

**Affiliations:** ^1^CAS Center for Excellence in Nanoscience, National Center for Nanoscience and Technology, Beijing, China; ^2^National Center for Nanoscience and Technology, University of Chinese Academy of Sciences, Beijing, China

**Keywords:** membrane tension, atomic force microscopy, cell mechanics, cytoskeleton network, cancer cells

## Abstract

Differences in stiffness constitute an extremely important aspect of the mechanical differences between cancer cells and normal cells, and atomic force microscopy (AFM) is the most commonly used tool to characterize the difference in stiffness. However, the process of mechanical characterization using AFM has been controversial and the influence of the membrane tension on AFM measurement results was often ignored. Here, a physical model involving a simultaneous consideration of the effects of the cell membrane, cytoskeleton network and cytosol was proposed. We carried out a theoretical analysis of AFM force relaxation curves, and as a result solved many of the remaining controversial issues regarding AFM-based mechanical characterization of cells, and provided a quantitative solution for the membrane tension measured using AFM indentation experiments for the first time. From the results of experiments on cells with different adherent shapes and different pairs of normal cells and cancer cells, we found additional force provided by membrane tension to be the main component of the force applied to the AFM probe, with decreased cell membrane tension being the essential reason for the greater softness of cancer cells than of normal cells. Hence, regulating membrane tension may become an important method for regulating the behavior of cancer cells.

## Introduction

Whether for distinguishing the mechanical differences between normal cells and cancer cells (Cross et al., 2007), or for studying the responses of cells to the physical properties of substrates (Discher et al., 2005), stiffness has always been an important parameter in the study of cell mechanics. Atomic force microscopy (AFM) is one of the most commonly used methods to characterize cell stiffness, but the choice of the physical model in the process of AFM force curve analysis has been controversial. The Hertz-Sneddon model (Hertz, 1882; Sneddon, 1965) is the most commonly used method which treated cells as elastic materials to analyze AFM force curves and characterize cell stiffness with the Young’s modulus ***E_Y_***. However, just as Darling (Darling et al., 2006) and Moeendarbary (Moeendarbary et al., 2013) tried to treat cells as viscoelastic materials and porous media, the Hertz-Sneddon theory is not very suitable to the actual situation of cells, and the cell stiffness calculated using the Hertz-Sneddon model has been found to always decrease with increasing indentation (Pogoda et al., 2012) and to increase with increasing loading rate (Li et al., 2008). Moreover, stiffness of living cells measured using AFM is always much higher than that measured using other methods (Ding et al., 2018) such as micropipette aspiration (Hochmuth, 2000) and particle-tracking microrheology (Wirtz, 2009). These inconsistent results may be a result of the use of inappropriate physical models, and hence it is of urgent importance to develop an appropriate physical model for analyzing AFM force curves.

The aforementioned elastic model, viscoelastic model and poroelastic model for AFM characterization always treat cells as homogeneous and isotropic bodies. However, there is a hierarchical structure in the cell, and the mechanical properties of the cell may be anisotropic (Efremov et al., 2019). In the process of analyzing cell indentation experiments with the above-mentioned theories, previous researchers often regarded cells as homogeneous bodies and focused on the mechanical behavior of the cytoplasm, while ignoring the influence of surface tension provided by the cell membrane (Ding et al., 2018). But recent studies have shown that surface tension may play a relatively important role in cell elasticity derived from AFM force curves (Ding et al., 2018), and have highlighted the importance of the cell membrane in cell mechanical behavior. The mechanical properties of the cell membrane are particularly important (Sitarska and Diz-Munoz, 2020). Cell membrane tension is involved in the regulation of membrane transport and the local curvature of the cell (Masters et al., 2013; Prévost et al., 2015), cell migration (Raucher and Sheetz, 2000), and cell polarity (Tsujita et al., 2015). A large number of studies have shown that there are various differences between the cell membranes of cancer cells and normal cells, such as the number and composition of membrane proteins (Liang et al., 2006; Li et al., 2012) and carbohydrates (Chen et al., 2016). The membrane diversity may cause differences in the cell membrane tension (Sitarska and Diz-Munoz, 2020), leading to a series of changes in cell membrane functions (Prévost et al., 2015) such as cell membrane permeability (Van der Paal et al., 2016) and repair capabilities (Frandsen et al., 2016). Wang et al. showed for the first time the ability to inhibit metastasis of cancer cells by increasing the tension of the cell membrane (Wang et al., 2020), suggesting broad application prospects for manipulations of membrane tension in tumor treatment. However, current methods used to quantify membrane tension with AFM are mainly based on measuring the force required to pull and hold a plasma membrane tube (also called a tether) (Diz-Munoz et al., 2018; Sitarska and Diz-Munoz, 2020), while directly deriving surface tension values from such measurements is challenging. Therefore, it is important to develop a quantitative solution for the relationship between surface tension and an AFM force curve and to understand the specific role of cell membrane tension in AFM indentation experiments.

Besides, a lot of studies have proved that the cytoskeleton is oriented (Pomp et al., 2018), which may lead to anisotropy of cell structure. The anisotropy of cytoskeleton not only controls the shape and force of the adherent cells (Schakenraad et al., 2020), but also affects the biological behaviors of living cells (Wei et al., 2020). Different cells may show varying degrees of anisotropy, which will also affect the results of cell characterization (Efremov et al., 2019). So, analyzing the anisotropic mechanical properties of the cytoskeleton with AFM characterization will be of great significance to mechanobiological features studies of cells.

In the current work, we developed a physical model designed to take into account the surface tension of the cell membrane, the elasticity of the cytoskeleton network, and the viscoelasticity of the cytosol simultaneously, and obtained for the first time a quantitative way to derive surface tension of a cell membrane from an AFM force relaxation curve. We also carried out a theoretical analysis of the influences of indentation depth and loading rate on the Young’s modulus ***E_Y_*** measured using the traditional method, and carried out a comparative analysis of the difference between the physical models used for the different cell stiffness measurement methods; the results of these analyses combined with the corresponding finite element simulation results by Ding et al. showed the advanced nature and high accuracy of our new physical model. By comparing cells with different adherent shapes and different normal-cancer cell pairs, we found cytoskeleton networks with different structures show varying elasticities, the main force in the AFM indentation experiments to be provided by the cell membrane surface tension rather than by the elasticity of the cytoskeleton network, and that changes in the surface tension of the cell membrane may be the essential cause of the decrease in stiffness that occurs when cells become cancerous. We therefore consider that changing the mechanical behavior of cancer cells by changing the membrane tension is a promising method for treating cancer.

## Materials and Methods

### Cell Culture

L929 mouse fibroblasts, 4T1 mouse breast cancer cells, and MCF-7 human breast cancer cells were obtained from the Laboratory for Biological Effects of Nanomaterials and Nanosafety of the National Centre for Nanoscience and Technology (NCNST). HC11 mouse mammary epithelial cells were kindly provided by Stem Cell Bank, Chinese Academy of Sciences. MCF-10A human normal mammary epithelial cells (Procell CL-0525) were kindly provided by Procell Life Science & Technology Co., Ltd.

L929, HC11, and 4T1 cells were cultured in Roswell Park Memorial Institute (RPMI-1640) medium containing 10% fetal bovine serum (FBS) with 1% penicillin/streptomycin (P/S) and 0.5% L-glutamine. MCF-7 cells were grown in Dulbecco’s modified Eagle’s medium (DMEM) containing 10% FBS with 1% P/S and 0.5% glutamine. MCF-10A cells were cultured in DMEM/F12 plus 5% horse serum (HS) supplemented with 20 ng/mL epidermal growth factor, 0.5 μg/mL hydrocortisone, 10 μg/mL insulin, 1% non-essential amino acids and 1% P/S. All cells were cultured at 37^*o*^C in an atmosphere of 5% CO_2_ in air.

Some experiments on culturing the same kind of cells in different medium also have been designed to observe the differences in their physical properties. For details, see Supplementary Results.

### Cell Preparation for AFM Experiments

In order to reduce the changes in the mechanical properties of cells during the culture process (Dokukin et al., 2017), cells between passage 6 and passage 10 were used for experiments. The cells were seeded in 35 × 10 mm Petri dish (Corning). Each experiment was started 12 h after cell passage. Right before the experiment, all samples were gently rinsed with serum-free medium (RPMI-1640 for L929, HC11, and 4T1, DMEM for MCF-7, and DMEM/F12 for MCF-10A) to remove possible detached cells, and left for 30 min in the same serum-free medium in the incubator.

### Atomic Force Microscopy Force Curve Acquisition

An AFM (Agilent 5500) with an inverted microscope (Nikon Eclipse Ti) was used to observe and locate the relative positions of the probe and cell, and obtain the force curves. In our experiments, an AFM probe consisting of a silicon nitride tipless cantilever (TL-CONT, Nanosensors) and coupled to 10-μm-diameter SiO_2_ particles with experimentally determined spring constants was used to capture the force curve. The spring constant of the AFM probe was experimentally determined to be 0.095 N/m.

**FIGURE 1:**
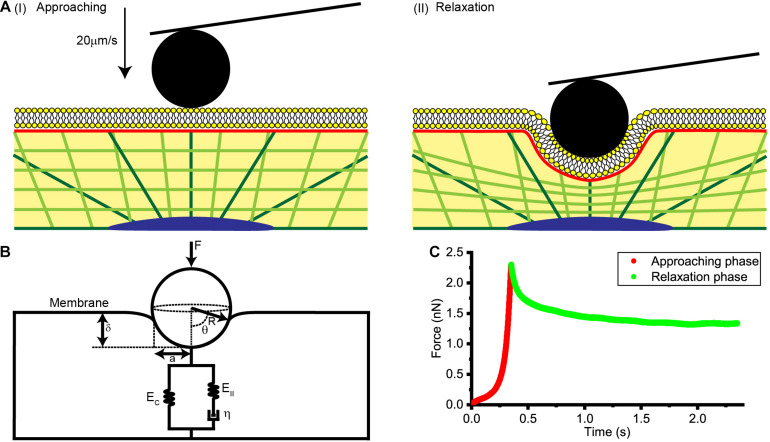
Collection and analysis of AFM force relaxation curves. **(A)** Schematic diagrams of the process used to acquire the AFM force relaxation curves. **(I)** Diagram showing the AFM probe approaching the cell at a speed of 20 μm/s. **(II)** Diagram showing the AFM probe making an indentation of about 1 μm in the cell and then kept still at this location. **(B)** A physical model of the cell membrane, cytoskeleton, and cytosol considered together for a spherical probe. **F** is the loading force of AFM indenter, **R** is the radius of spherical probe, θ is the integral angle, δ is indentation depth, **a** is the effective contact radius, **E_C_**, **E_II_**, and η are the elastic and viscos elements that make up the standard linear solid model of cytoplasm. **(C)** Force-time curve at the approaching phase and relaxation phase.

As is shown in Figure 1A, AFM tips were made to approach the cell surface at a rate of 20 μm/s, produce an indentation with a depth of about 1 μm, and then to remain stationary for about 3 s until the force relaxation signal gradually stabilized. Distance, time and deflection data for the whole process were recorded and used in subsequent theoretical calculations and data analysis.

Thirty cells for each type of cell morphology were selected. For each cell, three force curves were collected and averaged. The experiment of each type of cell was completed within 1 h.

### Theory for Calculating Cell Membrane Surface Tension and Cytoskeleton Elastic Modulus From a Force Relaxation Curve

Due to the effect of membrane tension, the pressure at the inner surface of a curved part of a cell membrane differs from that at its outer surface. When using a spherical probe, these measurements may be related using the equations

$${\text{F}}_{\mathrm{SP}}=\int {\text{P}}_{\mathrm{OUT}}\,\text{dS}$$

and

$${\text{P}}_{\mathrm{OUT}}={\text{P}}_{\mathrm{IN}}+\Delta \,\text{P},$$

where ***F_SP_*** denotes the indentation force of the AFM probe, ***P_IN_*** and ***P_OUT_*** the pressures of the contact areas inside and outside the membrane, respectively, Δ***P*** the additional pressure provided by membrane tension, and ***S*** the area of contact of the spherical probe with the cell membrane.

Combining these equations yields the equation

$$F_{SP}=\;\int^{\text{​}}(P_{IN}+\Delta P)dS\;=\;\int^{\text{​}}P_{IN}dS+\int^{\text{​}}\Delta PdS\;=F_{SP-C}+F_{SP-M},$$

where ***F**_**SP**−**C**_* and ***F**_**SP**−**M**_* denote the viscoelastic force provided by the cytoplasm and the additional force provided by the membrane tension, respectively. Due to the thickness of any cell membrane being much smaller than the size of the whole cell, the elastic force provided by the cell membrane was ignored.

For ***F**_**SP**−**M**_*, and considering that changes in membrane tension were previously shown to not propagate over long distances in the plasma membrane (Shi et al., 2018), we assumed no change occurring in the cell membrane surface tension γ during the AFM indentation experiment, and assumed the curvature radius of the membrane surface to be the same as that of the spherical probe. According to Laplace’s equation, the additional pressure Δ***P*** provided by a membrane is

$$\Delta \,\text{P}=\frac{2\,\gamma }{\text{R}},$$

where γ denotes the membrane tension, and ***R*** denotes the radius of the spherical probe.

Considering the symmetry of the spherical probe yields the equation

$${\text{F}}_{\mathrm{SP}-M}=\int \Delta \,\text{PdScos}\,\theta ,$$

where θ denotes the angle shown in Figure 1B.

The force provided by membrane can be calculated by

$${\text{F}}_{\mathrm{SP}-M}=\int_{0}^{\theta }\Delta \,\text{P2}\,\pi \,\text{Rsin}\,\theta \,\text{Rcos}\,\theta \,d\theta =\Delta \,\text{P}\,\pi \,{\text{R}}^{2}\,{\text{sin}}^{2}\,\theta .$$

According to Sneddon (1965), the radius ***a*** of the effective contact area (Figure 1B) is $\sqrt{\text{R}\,\delta }$, leading to a relationship between indentation depth δ, ***R***, and θ as described by the equation

$$\text{sin}\,\theta =\frac{\text{a}}{\text{R}}=\frac{\sqrt{\text{R}\,\delta }}{\text{R}}.$$

According to Moeendarbary et al. (2013), the change in the indentation depth δ during relaxation phase is negligible, and the Poisson’s ratio ***v*** of a cell may be taken as 0.3.

So ***F**_**SP**−**M**_* can be expressed as

$${\text{F}}_{\mathrm{SP}-M}={\text{F}}_{\mathrm{SP}-M}\,\left(t\right)=\Delta \,\text{P}\,\pi \,\text{R}\,\delta =2\,\gamma \,\pi \,\delta ,$$

where ***F**_**SP**−**M**_*(***t***) denotes the force of the interaction between the membrane and spherical probe at time ***t*** of the force relaxation phase.

For ***F**_**SP**−**C**_*, we considered the cytoplasm as a standard linear solid consisting of a spring (stiffness of cytoskeleton ***E_C_***) in parallel with a spring-dashpot (apparent stiffness ***E_II_*** and apparent viscosity η of the cytosol). So based on the theory of Darling et al. (2006), we derived a formula for force relaxation when the cytoplasm is pressed by a spherical probe, with this formula expressed as

$${\text{F}}_{\mathrm{SP}-C}\,\left(t\right)=\frac{4}{3}\,\frac{{\text{R}}^{\frac{1}{2}}\,\delta^{\frac{3}{2}}\,{\text{E}}_{C}}{1-{\text{ν}}^{2}}\,\left(1+\frac{\tau_{\sigma }-\tau_{\varepsilon }}{\tau_{\varepsilon }}\,{\text{e}}^{-\frac{t}{\tau_{\varepsilon }}}\right),$$

where ***F**_**SP**−**C**_*(***t***) denotes the force of the interaction between the cytoplasm and spherical probe at time ***t*** of the force relaxation phase, ***E_C_*** denotes the elastic modulus of the cytoskeleton network, τ_σ_ and τ_ε_ denote the relaxation times under constant load and deformation, respectively, and η = ***E_C_***(τ_σ_−τ_ε_).

The indentation force of a spherical probe at time ***t***, i.e., ***F_SP_***(***t***), was derived based on the above theories to be

$$F_{SP}\left(t\right)\;=F_{SP-M}+F_{SP-C}\left(t\right)\;=2\gamma \pi \delta +\;\frac{4}{3}\frac{R^{\frac{1}{2}}\delta^{\frac{3}{2}}E_{C}}{1-\nu^{2}}\left(1+\frac{\tau_{\sigma }-\tau_{\varepsilon }}{\tau_{\varepsilon }}e^{-\frac{t}{\tau_{\varepsilon }}}\right).$$

Based on the above conclusions, we found that our theory is consistent with the theory of Ding et al. Ding et al. (2018) found an interesting phenomenon when using dimensional analysis and finite element simulation to analyze traditional AFM force-distance curves for considering the effect of the cell membrane – that is,

$$\frac{\left({\text{F}}_{\mathrm{SP}}-{\text{F}}_{\mathrm{SP}-C}\right)}{{\text{F}}_{\mathrm{SP}-C}}\propto \frac{2\,\gamma }{{\text{E}}^{*}\,\sqrt{\text{R}\,\delta }}$$

for a spherical probe, where *E*^∗^ denotes the apparent stiffness of the cytoplasm. According to this interesting phenomenon, they proposed an equation considering AFM indentation force of the cell membrane (Ding et al., 2018). However, their work only discovered this phenomenon but could not explain it theoretically.

In contrast, we were able to explain the occurrence of this phenomenon using our model and formula. According to our theory as described above,

$$\frac{\left({\text{F}}_{\mathrm{SP}}\,\left(0\right)-{\text{F}}_{\mathrm{SP}-C}\,\left(0\right)\right)}{{\text{F}}_{\mathrm{SP}-C}\,\left(0\right)}=\frac{3\pi \left(1-\nu {}^{2}\right)\tau_{\varepsilon }}{4\,\tau_{\sigma }}\,\frac{2\,\gamma }{{\text{E}}_{C}\,{\text{R}}^{\frac{1}{2}}\,\delta^{\frac{1}{2}}}.$$

This formula can explain the interesting phenomenon mentioned above and further prove the validity of our theory.

Next we will talk about how to calculate the elastic modulus of the cytoskeleton network ***E_C_*** and the membrane tension γ. Extrapolating ***F_SP_***(***t***) to an infinitely long time,

$${\text{F}}_{\mathrm{SP}}\,\left(\infty \right)=2\,\gamma \,\pi \,\delta +\frac{4}{3}\,\frac{{\text{R}}^{\frac{1}{2}}\,\delta^{\frac{3}{2}}\,{\text{E}}_{C}}{1-{\text{ν}}^{2}}$$

and

$${\text{F}}_{\mathrm{SP}}\,\left(t\right)-{\text{F}}_{\mathrm{SP}}\,\left(\infty \right)=\frac{4}{3}\,\frac{{\text{R}}^{\frac{1}{2}}\,\delta^{\frac{3}{2}}\,{\text{E}}_{C}}{1-{\text{ν}}^{2}}\,\frac{\tau_{\sigma }-\tau_{\varepsilon }}{\tau_{\varepsilon }}\,{\text{e}}^{-\frac{t}{\tau_{\varepsilon }}}.$$

Here, δ can be calculated by the force curve at approaching phase (Figure 1C), so by fitting the data with equation ***F_SP_***(***t***)−***F_SP_***(∞), we were able to calculate the elastic modulus of the cytoskeleton network ***E_C_*** and the relaxation times τ_σ_ and τ_ε_.

In this way,

$${\text{F}}_{\mathrm{SP}-M}={\text{F}}_{\mathrm{SP}}\,\left(\infty \right)-\frac{4}{3}\,\frac{{\text{R}}^{\frac{1}{2}}\,\delta^{\frac{3}{2}}\,{\text{E}}_{C}}{1-{\text{ν}}^{2}}$$

and the membrane tension may be calculated using the equation

$$\gamma =\frac{{\text{F}}_{\mathrm{SP}}\,\left(\infty \right)-\frac{4}{3}\,\frac{R^{\frac{1}{2}}\,\delta^{\frac{3}{2}}\,E_{C}}{1-\nu^{2}}}{2\,\pi \,\delta }.$$

Similarly, we derived the calculations for a conical probe (see Supplementary Theory).

### Data Analysis

Atomic force microscopy curves were analyzed with custom-written code in MATLAB (MathWorks Inc.). Approaching force-distance curves were used to calculate the contacting point and the indentation depth. Relaxation force-time curves were used to calculate the instantaneous modulus ***E_I_*** (equivalent to Young’s modulus ***E_Y_*** used with the traditional method), the relaxed modulus ***E_R_***, the cytoskeleton modulus ***E_C_***, the apparent viscosity of the cytosol η, the membrane tension γ, the force exerted by the membrane ***F**_**SP**−**M**_*, and the force provided by the cytoplasm ***F**_**SP**−**C**_*.

***E_C_***, γ, η, ***F**_**SP**−**M**_*, and ***F**_**SP**−**C**_* were calculated as described above. To compare these results with those of traditional measurement methods, the forces and indentations at the beginning and end of the relaxation phase (Figure 1C) were used to calculate the instantaneous modulus ***E_I_*** and the relaxed modulus ***E_R_*** according to the Hertz-Sneddon model. In this way, cell stiffness was characterized.

$$E_{I}=\;\;\frac{3}{4}\frac{\left(1-\nu^{2}\right)F_{SP}\left(0\right)}{R^{\frac{1}{2}}\delta^{\frac{3}{2}}}$$

$$E_{R}\;=\;\frac{3}{4}\frac{\left(1-\nu^{2}\right)F_{SP}\left(\infty \right)}{R^{\frac{1}{2}}\delta^{\frac{3}{2}}}$$

The apparent stiffness of the interstitial fluid ***E_II_*** was obtained from the relationships

$${\text{E}}_{I}={\text{E}}_{R}+{\text{E}}_{\mathrm{II}}$$

and

$${\text{E}}_{\mathrm{II}}={\text{E}}_{R}\,\left(\frac{\tau_{\sigma }-\tau_{\varepsilon }}{\tau_{\varepsilon }}\right).$$

Cell stiffness ***E_Y_*** measured using the traditional method always decreases with increasing indentation depth (Pogoda et al., 2012; Ding et al., 2018). Ding et al. found that if the effect of surface tension is considered, the elastic modulus of the cytoplasm will not depend on the indentation depth. We can theoretically prove that the instantaneous modulus ***E_I_*** (equals to ***E_Y_***) of the cell decreases as the indentation depth increases is an inevitable result. Comparing our formula with the traditional Hertz model yields

$${\text{F}}_{\mathrm{SP}}\,\left(0\right)=\frac{4}{3}\,\frac{{\text{R}}^{\frac{1}{2}}\,\delta^{\frac{3}{2}}}{\left(1-{\text{ν}}^{2}\right)}\,{\text{E}}_{I}=2\,\gamma \,\pi \,\delta +\frac{4}{3}\,\frac{{\text{R}}^{\frac{1}{2}}\,\delta^{\frac{3}{2}}\,{\text{E}}_{C}}{1-{\text{ν}}^{2}}\,\frac{\tau_{\sigma }}{\tau_{\varepsilon }}$$

and

$${\text{E}}_{I}=\frac{3\,\pi \,\gamma \,\left(1-{\text{ν}}^{2}\right)}{2\,{\text{R}}^{\frac{1}{2}}\,\delta^{\frac{1}{2}}}+{\text{E}}_{C}\,\frac{\tau_{\sigma }}{\tau_{\varepsilon }},$$

expressions showing a decreasing instantaneous modulus with increasing indentation depth.

For a conical probe, similar relationships can be obtained, details of which are provided in Supplementary Theory.

Statistical analysis was performed using Origin (OriginLab) and Statistical Product and Service Solutions (SPSS) (IBM). The calculated value for each group of variables is presented in terms of mean value and standard deviation (mean ± SD). The statistical significance of differences in mean values was assessed using a two-sample independent Student’s *t*-test at the 95% confidence level. ^∗^*p* < 0.05, ^∗∗^*p* < 0.01, ^∗∗∗^*p* < 0.001. No label indicates no significant difference between the two groups.

### Cell Staining and Confocal Imaging

TRITC-phalloidin (Solarbio) staining was used to detect fibrillary actin (F-actin). Medium was first pipetted out and rinsed twice with phosphate-buffered saline (PBS) for 10 min each time. Then, the cells were fixed with a 4% formaldehyde solution dissolved in PBS for 10 min. The fixed cells were rinsed with PBS twice for 10 min each time. The rinsed cells were treated with a 0.5% Triton x-100 solution for 5 min, and then rinsed twice with PBS for 10 min each time. A volume of 200 μL of 100 nM TRITC-phalloidin was added to the resulting cells, and the cells were then incubated in the dark for 30 min. Then, the cells were rinsed with PBS three times for 5 min each time. Finally, a volume of 200 μL of Fluoroshield medium containing DAPI (Sigma) was added to counterstain the nucleus and prevent the fluorescence from quenching. The corresponding images were captured by using an UltraVIEW VoX (Perkin Elmer) spinning disk confocal unit.

### Finite Element Simulation

**FIGURE 2:**
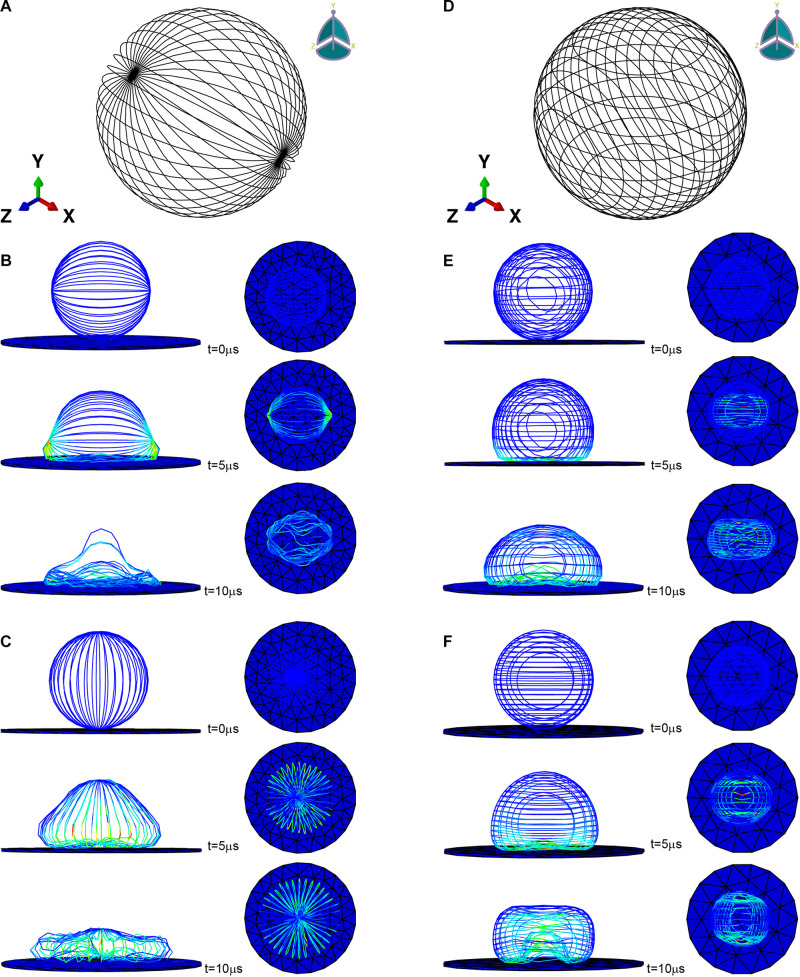
Microfilament network model of **(A)** L929 and **(D)** HC11. **(B,C)** are the adherence process when the microfilament network model of L929 is subjected to gravity toward the *Y* axis and toward the *X* axis, respectively. **(E,F)** are the adherence process when the microfilament network model of HC11 is subjected to gravity toward the *Y* axis and toward the *Z* axis, respectively.

ABAQUS (SIMULIA) was used to implement the finite element simulation. The initial shape of the microfilament network was modeled as hollow sphere with a radius of 20 μm composed of beam elements (used to simulate microfilament bundles) (Figures 2A,D). For microfilament, the mass density was set to 10^3^*kg*/m^3^ (Zeng and Li, 2011), the Young’s modulus was set to 1.4 GPa (Gittes et al., 1993), and the Poisson’s ratio was set to 0.3. The cross-sectional shape of the beam element was set as a circle with a radius of 50 nm (estimated from the stained image). The substrate was modeled as a cylinder glass with a radius of 100 μm and a thickness of 1 μm, and the mass density was set to 2.5×10^3^*kg*/m^3^, the Young’s modulus was set to 88 GPa, the Poisson’s ratio was set to 0.3. In order to speed up the adhesion process and shorten the time required for the finite element simulation, the gravity was magnified and set to 9.8×10^5^m/s^2^ (Zeng and Li, 2011).

## Results

### Different Cytoskeleton Network Structures of Cells With Different Adherent Morphologies

**FIGURE 3:**
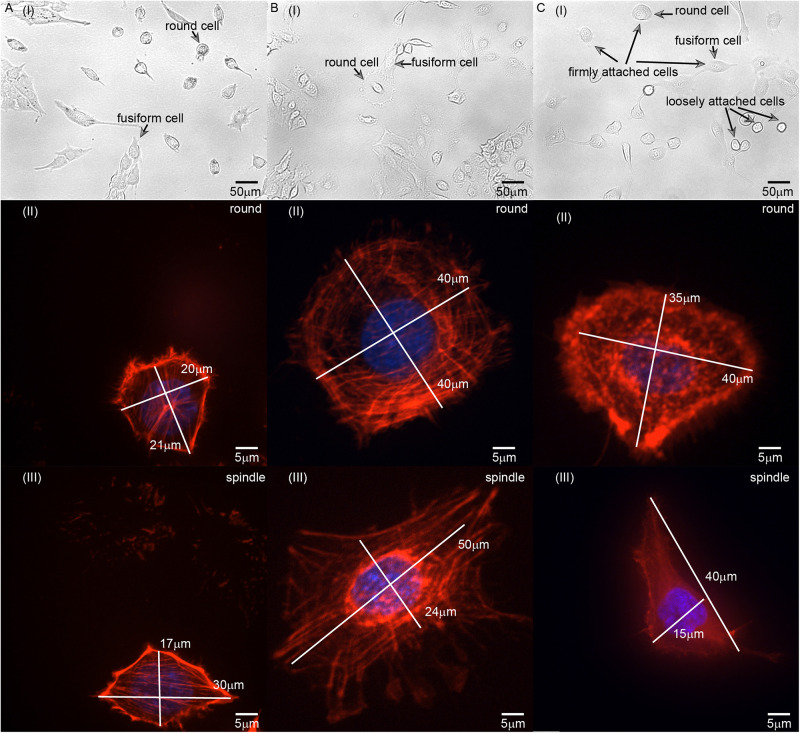
Images of **(A)** L929, **(B)** HC11, and **(C)** MCF-10A cells with different adherent shapes and acquired using optical microscopy and confocal microscopy. **[A(I)–C(I)]** are the images captured by optical microscope. Examples of round and fusiform cells, loosely attached and firmly attached cells have been marked separately in the figure. **[A(II)–C(II)]** are the images of round cells captured by laser scanning confocal microscope. **[A(III)–C(III)]** are the images of fusiform cells captured by laser scanning confocal microscope. The length of the long and short axis of cells has also been marked in **[A(II–III)–C(II–III)]**.

L929 is a facultative adherent cell, and it often forms both round and fusiform shapes in the adherent state (Figure 3A). According to published descriptions and characterizations of the cytoskeleton, microtubules are mainly distributed around the nucleus (Dogterom and Koenderink, 2019), intermediate fibers are distributed in a disordered manner in the whole cell (Bertaud et al., 2010), and microfilaments are mainly distributed on the inner sides of cells and always adopt a certain orientation (Li et al., 2020). The microfilaments are usually connected to the cell membrane through cytoskeleton connexin (for example, Ezrin) (Sitarska and Diz-Munoz, 2020), and it may affect the tension of the cell membrane and the shape of the cell. Therefore, we focused on the relationship between microfilaments and cell morphology.

We also set out to compare the properties of normal HC11 and MCF-10A breast cells. Inspection of optical microscopy and laser confocal microscopy images of these cells showed for the most part different fusiform and round shapes formed by L929, HC11, and MCF-10A cells (Figure 3). The classification of round and fusiform cells was qualitative first. According to the classification and comparison of the images collected in the experiment, it can be summarized that fusiform cells and round cells were classified according to whether the ratio of the long and short axis of the cell is greater than 1.3. It’s worth noting that there are both large and small round cells. According to the literature of previous researchers, the small round cells may adhere to the substrate loosely but the large round cells can adhere to the substrate firmly (Dokukin et al., 2017) [Figure 3C(I)]. In this article, all the cells selected for the experiments were firmly attached. We also observed the cytoskeletal network structure of cells with different shapes to compare their anisotropy. For L929 and MCF-10A cells, the cytoskeleton network structure of the fusiform cells was observed to differ obviously from that of the round cells – with the microfilament orientation of the round cells perpendicular to the adhesion plane, and the microfilaments of the fusiform cells arranged along the cellular long axis (Figures 3A,C). For HC11 cells, the cytoskeleton structures of the fusiform cells differed in some respects but were similar in others to those of the round cells: they were both observed to be composed of round and linear microfilaments as is shown in Figure 3B. As shown in Figures 2A,D, we constructed different finite element models according to the observed structure of the cytoskeletal networks.

Previous studies have proved that the orientation of the cytoskeleton has a certain correlation with the shape of the cell (Pomp et al., 2018; Schakenraad et al., 2020). For part of the cells mentioned in this section, we have also conducted a study on the correlation between the structure of the microfilament network and the shape after attachment. Figures 2A,D are the microfilament model diagrams established based on the staining results of L929 and HC11 cytoskeleton, respectively. Figures 2B,C, respectively show the adherence process when the microfilament network model in Figure 2A is subjected to gravity toward the *Y* axis and toward the *X* axis. Figures 2E,F, respectively show the adherence process when the microfilament network model in Figure 2D is subjected to gravity toward the *Y* axis and toward the *Z* axis. It can be seen from Figure 2 that microfilament networks with different structures have varying shapes after adherence, and microfilament networks with the same structure will have diverse shapes after adherence under the action of gravity in different directions due to their anisotropy.

### Major Role of the Cell Membrane in Cell Stiffness According to a Comparison of the Mechanical Parameters of the Same Cell With Different Adherent Shapes

The physical model we proposed above indicated the cell membrane, cytoskeleton, and cytosol all contribute to the instantaneous modulus ***E_I_*** (which is equivalent to the Young’s modulus ***E_Y_*** measured by traditional method) but only the cell membrane and cytoskeleton contribute to the relaxed modulus ***E_R_***. Our model and other data indicated an effect of the orientation of the microfilaments on the morphology of cells after adhesion – with the altered cell morphology suggesting an altered mechanical environment imposed on the cytoskeleton network, and hence an altered structure and stiffness of the cytoskeleton network, altered interaction between the cytosol and cytoskeleton, and thus altered viscoelasticity behavior of the cytosol in the relaxation process.

**FIGURE 4:**
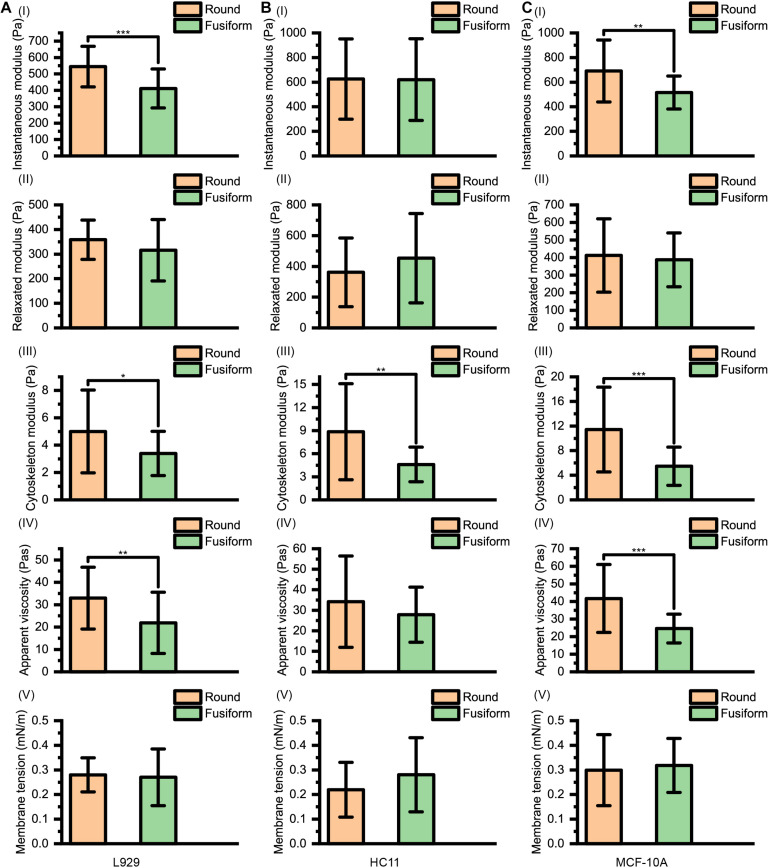
Instantaneous modulus, relaxed modulus, cytoskeleton modulus, apparent viscosity, and membrane tension values for **(A)** L929, **(B)** HC11, and **(C)** MCF-10A cells with different adherent shapes.

The instantaneous modulus ***E_I_***, the relaxed modulus ***E_R_***, the cytoskeleton modulus ***E_C_***, the apparent viscosity of cytosol η and the membrane tension γ of L929, HC11 and MCF-10A cells with different shapes are shown in Figure 4. We noticed that for the cells (L929 and MCF-10A) with very large differences in the arrangement of the cytoskeleton network at different adherent morphologies, significantly different instantaneous modulus values were observed (L929: 544.72 ± 124.06 Pa for round cells and 410.98 ± 118.78 Pa for fusiform cells, *p* < 0.001; MCF-10A: 690.43 ± 252.15 Pa for round cells and 516.08 ± 133.95 Pa for fusiform cells, *p* = 0.001) but the relaxation modulus showed no significant difference (L929: 358.56 ± 79.93 Pa for round cells and 315.73 ± 124.85 Pa for fusiform cells, *p* = 0.119; MCF-10A: 412.12 ± 208.83 Pa for round cells and 387.52 ± 153.04 Pa for fusiform cells, *p* = 0.604). This result differed from the cells (HC11) displaying similar cytoskeletal networks at different adherent morphologies: the round and fusiform HC11 cells showed instantaneous modulus values not significantly different from one another (625.23 ± 325.80 Pa for round cells and 620.56 ± 332.03 Pa for fusiform cells, *p* = 0.956), as was also the case for the relaxation modulus values (361.38 ± 223.32 Pa for round cells and 453.14 ± 290.22 Pa for fusiform cells, *p* = 0.175). The calculated instantaneous modulus was often found to be about 200 Pa higher than the relaxed modulus, due to the relaxation modulus not including the instantaneous modulus provided by the cell cytosol. The cell stiffness ***E_Y_*** measured using the traditional method was shown to be equivalent to the instantaneous modulus **E_I_** of the cell, but the apparent stiffness of the cytosol is easily affected by the loading rate of the AFM indenter, thus explaining why the cell stiffness measured using traditional methods is also easily affected by the loading rate. We considered the relaxed modulus ***E_R_*** to be more suitable than instantaneous modulus ***E_I_*** to characterize the cell stiffness and to eliminate the difference in measurement results caused by different loading rates. Specifically, using ***E_R_*** as the standard of cell stiffness was expected to eliminate any effect of the loading rate on the measurement of cell stiffness.

The apparent viscosity of the cytosol (L929: 32.96 ± 13.80 Pa⋅s for round cells and 21.86 ± 13.66 Pa⋅s for fusiform cells, *p* = 0.003; HC11: 34.22 ± 22.26 Pa⋅s for round cells and 27.84 ± 13.47 Pa⋅s for fusiform cells, *p* = 0.184; MCF-10A: 41.69 ± 19.36 Pa⋅s for round cells and 24.63 ± 8.19 Pa⋅s for fusiform cells, *p* < 0.001) also showed a dependence on the structure of the cytoskeleton network, attributed to the apparent viscosity of the cell cytosol affected by both the structure of cytoskeleton network and the viscosity of the cell cytosol (Moeendarbary et al., 2013).

For these three kinds of cells, the relaxed modulus ***E_R_*** was found to not depend on the morphology of the adherent cell. But for each kind of cell, the cells with different adherent shapes showed significantly different cytoskeleton network modulus values (L929: 5.00 ± 3.03 Pa for round cells and 3.40 ± 1.62 Pa for fusiform cells, *p* = 0.013; HC11: 8.86 ± 6.25 Pa for round cells and 4.61 ± 2.26 Pa for fusiform cells, *p* = 0.001; MCF-10A: 11.43 ± 6.90 Pa for round cells and 5.47 ± 3.11 Pa for fusiform cells, *p* < 0.001) but no significant difference in surface membrane tension values (L929: 0.28 ± 0.07 mNm^–1^ for round cells and 0.27 ± 0.12 mNm^–1^ for fusiform cells, *p* = 0.693; HC11: 0.22 ± 0.11 mNm^–1^ for round cells and 0.28 ± 0.15 mNm^–1^ for fusiform cells, *p* = 0.079; MCF-10A: 0.30 ± 0.14 mNm^–1^ for round cells and 0.32 ± 0.11 mNm^–1^ for fusiform cells, *p* = 0.565). These results suggested that the cytoskeleton may not be the main factor affecting cell stiffness. The force provided by the cytoskeleton network, i.e., **F**_**SP**−**C**_(∞), was found to be less than 5% of that provided by the cell membrane, i.e., **F**_**SP**−**M**_(Table 1), indicating the decisive role played by the cell membrane rather than by the cytoskeleton network in the characterization of cell stiffness.

**TABLE 1:** Values of force applied to an AFM probe after relaxation *F*_*SP*_(∞), force provided by the membrane *F*_*SP–M*_ and force provided by the cytoskeleton network *F*_*SP*−*C*_(∞) for cells with different adherent shapes.

	F_SP_(∞) (nN)	F_SP−M_ (nN)	F_SP−C_(∞) (nN)	F_SP−C_(∞)/F_SP−M_ (%)
L929-round	3.65	3.52	0.13	3.79
L929-fusiform	4.06	3.95	0.10	2.58
L929-all	3.85	3.74	0.11	3.01
HC11-round	1.86	1.79	0.07	4.00
HC11-fusiform	2.37	2.29	0.08	3.54
HC11-all	2.11	2.04	0.08	3.74
4T1	1.04	1.00	0.05	4.84
MCF-10A-round	4.52	4.32	0.20	4.62
MCF-10A-fusiform	5.76	5.60	0.16	2.86
MCF-10A-all	5.13	4.96	0.18	3.53
MCF-7	3.77	3.70	0.07	1.98

### Association of Changes in the Membrane Tension With a Reduction in the Stiffness of Cancer Cells According to a Comparison of the Mechanical Parameters of Normal and Cancer Cells

The cytoskeleton has long been considered to be the main factor affecting cell stiffness (Calzado-Martin et al., 2016), and investigators often try to change cell stiffness by regulating actin microfilaments (Gu et al., 2018). Due to cancer cells being softer than normal cells (Cross et al., 2007), some researchers have tried to adjust the stiffness of the cells by altering the actin filaments, and in this way combat cancer (Sun et al., 2017; Raudenska et al., 2019).

However, previous results have shown a decisive role played by membrane tension rather than by the cytoskeleton in the characterization of cell stiffness – and hence leaves the actual cause of the decreased cell stiffness of cancer cells yet to be determined. In order to compare the changes of mechanical properties of normal cells and cancer cells, we selected normal and cancerous breast cells from mouse and human sources, respectively.

**FIGURE 5:**
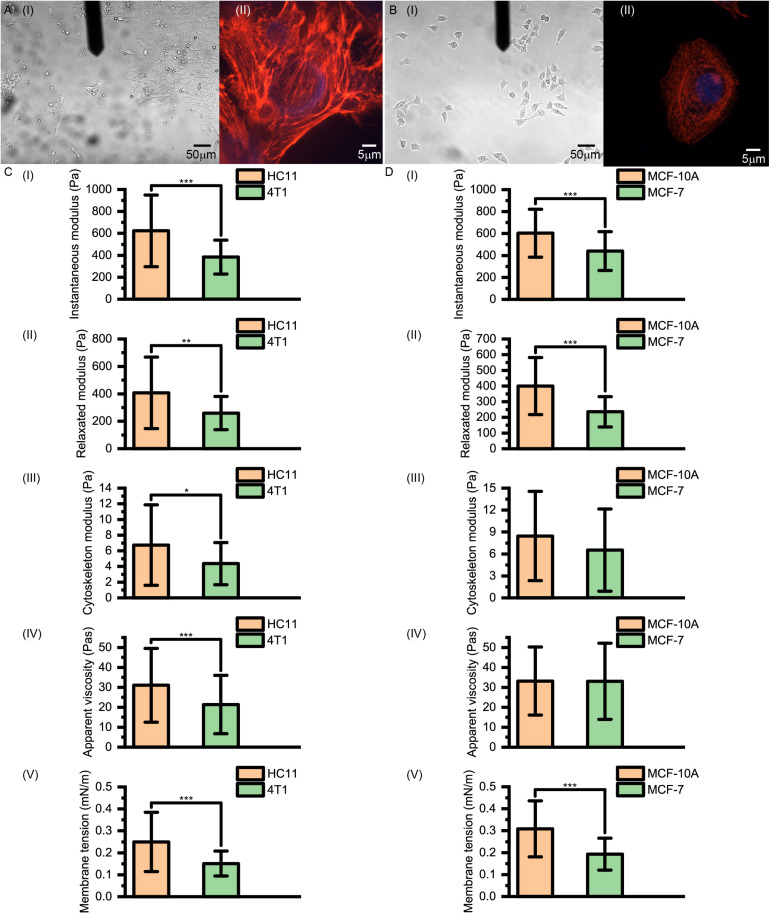
**(A)** Images of 4T1 and **(B)** MCF-7 cells acquired using optical microscopy and confocal microscopy. **(C,D)** Instantaneous modulus, relaxed modulus, cytoskeleton modulus, apparent viscosity and membrane tension values of **(C)** murine HC11 and 4T1 cells and **(D)** human MCF-10A and MCF-7 cells. HC11 and MCF-10A are normal breast cells and 4T1 and MCF-7 are cancerous breast cells.

As shown in Figure 5, for normal HC11 and MCF-10A breast cells and cancerous 4T1 and MCF-7 breast cells, the instantaneous modulus (HC11: 622.90 ± 326.14 Pa, 4T1: 383.87 ± 155.12 Pa, *p* < 0.001; MCF-10A: 603.26 ± 218.62 Pa, MCF-7: 440.20 ± 177.24 Pa, *p* < 0.001) and relaxation modulus (HC11: 407.26 ± 260.87 Pa, 4T1: 259.78 ± 121.26 Pa, *p* = 0.004; MCF-10A: 399.86 ± 181.94 Pa, MCF-7: 235.70 ± 96.55 Pa, *p* < 0.001) of the cancer cells were significantly higher than those of the normal cells. These results indicated that these cancer cells, while not complete elastomers, were indeed softer than the normal cells.

However, there is not necessarily a significant difference between the cytoskeleton network modulus values (HC11: 6.73 ± 5.13 Pa, 4T1: 4.37 ± 2.69 Pa, *p* = 0.020; MCF-10A: 8.45 ± 6.10 Pa, MCF-7: 6.53 ± 5.63 Pa, *p* = 0.098) and the apparent viscosity values of the cytosol (HC11: 31.03 ± 18.52 Pa⋅s, 4T1: 21.36 ± 14.64 Pa⋅s, *p* < 0.001; MCF-10A: 33.16 ± 17.06 Pa⋅s, MCF-7: 33.01 ± 19.10 Pa⋅s, *p* = 0.830) of normal cells and cancer cells, but their membrane tension values (HC11: 0.25 ± 0.13 mNm^–1^, 4T1: 0.15 ± 0.06 mNm^–1^, *p* < 0.001; MCF-10A: 0.31 ± 0.13 mNm^–1^, MCF-7: 0.19 ± 0.07 mNm^–1^, *p* < 0.001) did differ significantly. These cytoskeleton networks and membrane tension results once again suggested a correlation between cell membrane tension and instantaneous modulus. Our theoretical calculations suggested the force provided by the cytoskeleton network to be less than 5% of that provided by cell membrane surface tension (Table 1). These results indicated the softening of cancer cells, relative to normal cells, to be mostly due to decreases of surface tension – and highlighted the relative importance of differences in the cell membrane between cancer cells and normal cells. Thus, when aiming to change the cell stiffness, it might be more beneficial to work on adjusting the surface stiffness of the cell membrane than working on adjusting the cytoskeleton.

## Discussion

The characterization of cell mechanical properties using AFM has always been controversial. First, the cell does not fully satisfy the basic hypothesis of the Hertz model (Hertz, 1882). Secondly, the cell stiffness measured using AFM has been experimentally shown to be easily affected by the loading rate (Li et al., 2008) and indentation depth (Pogoda et al., 2012). Thirdly, cell stiffness values measured using AFM (Vargas-Pinto et al., 2013), micropipettes (Hochmuth, 2000) and particle-tracking microrheology (Wirtz, 2009) have been found to not be on the same order of magnitude, but instead to show values of several thousand Pascals, several hundreds of Pascals, and several tens of Pascals, respectively. Fourthly, despite being an important part of cells, the cell membrane is seldom considered in AFM measurements (Ding et al., 2018). Finally, AFM measurements of cell membrane surface tension only yield the tether force, and not the surface tension directly (Sun et al., 2005).

Although investigators have used various physical models to explain the mechanical behavior of cells in order to make the theoretical mechanical response as close as possible to the actual mechanical behavior of cells, there is still no theory that takes into account the effects of the cell membrane, cytoskeleton network and cytosol at the same time. By taking these three parts into account at the same time in this study, we were able to show that the considerable effect of loading rate on cell stiffness measured using the traditional method may be caused by the interaction between the viscoelastic cytosol and the cytoskeleton network – and using the relaxed modulus ***E_R_*** to characterize the cell stiffness would result in the measurement of the cell stiffness no longer being affected by the loading rate. In addition, we also gave the relationship between the instantaneous modulus and the indentation depth based on the theory of this article.

Our theory can also explain the observation of diverse cell modulus values being measured when different methods are used. Differences in the measurement results of different characterization techniques limit direct comparisons between datasets and may slow down the clinical application of cell mechanics instruments (Wu et al., 2018). Understanding the reasons for these differences will help promote the application of cell mechanics in clinical medicine. When using the traditional AFM method to measure cell stiffness, the force applied to the AFM probe is the sum of the forces from the cell membrane, cytoskeleton network, and cytosol, so the measured stiffness is equivalent to the instantaneous modulus ***E_I_***. When using a micropipette, the stiffness is measured in a stable state; here, the cell membrane and cytoskeleton play major roles in this condition, so the result of the measurement is equivalent to the relaxed modulus ***E_R_***. When using particle-tracking microrheology, the cytoskeleton network plays a major role, so the result of the measurement is equivalent to the cytoskeleton network modulus ***E_C_***. In short, our theory helps us understand the measurement process of various mechanical characterization methods.

More importantly, we overcame the previous challenges involving the quantitative characterization of cell membrane surface tension by AFM. Based on our theory and calculation results, the force provided by the cell membrane accounted for the majority of the total force exerted on the probe, while the force provided by the cytoskeleton network was less than 5% of the force provided by the cell membrane. From these results, the main culprit responsible for reducing the stiffness of cells during carcinogenesis may be the decrease of membrane tension. Consistent with the pioneering work of Wang et al. (2020), our research found that impeding the development of cancer by increasing cell membrane tension has a great future.

In summary, we developed and verified a physical model designed to consider the effects of the cell membrane, cytoskeleton and cytosol on AFM force relaxation curves. We not only explained theoretically why the Young’s modulus ***E_Y_*** measured using the traditional method is greatly affected by the loading rate and indentation depth, but also explained why different methods yield very different cell stiffness measurements. Most importantly, our theory was able to obtain for first time a quantitative determination of cell membrane tension from AFM force relaxation curves, and to show membrane tension may be the main source of the force detected by an AFM probe. Combined with previous studies on the structural and functional changes of cancer cell membranes compared to normal cell membranes, we believe that changes in cell membrane tension may be the main reason why cancer cells become soft. Our work suggests that there are broad prospects in regulating cell mechanical behavior and treating cancer by changing membrane tension.

## Data Availability Statement

The original contributions presented in the study are included in the article/Supplementary Material, further inquiries can be directed to the corresponding author.

## Author Contributions

KR: theoretical derivation, AFM experimental implementation, data analysis, MATLAB code writing, finite element simulation, and manuscript writing. JG: cell culture, AFM and cell staining experimental implementation, statistical analysis of data and images, finite element simulation, and manuscript writing and revision. DH: research supervision, manuscript critical review and revision. All the authors read and approved the final manuscript.

## Conflict of Interest

The authors declare that the research was conducted in the absence of any commercial or financial relationships that could be construed as a potential conflict of interest.
