# Supplementary material for: AFM Force Relaxation Curve Reveals That the Decrease of Membrane Tension Is the Essential Reason for the Softening of Cancer Cells
Source: Front Cell Dev Biol. 2021 May 12;9:663021. doi: 10.3389/fcell.2021.663021 (PMC8152666; doi:10.3389/fcell.2021.663021)
Supplement: Supplementary file 1 [file Data_Sheet_1.pdf]

Supplementary Material

1. Supplementary Theory

Use of a conical probe was analyzed (**Supplementary Figure 1**) in a manner similar to that used to analyze a spherical probe. Here, the indentation force of the AFM probe $\boldsymbol{F_{CO}}$ was calculated using the equation

$$\boldsymbol{F_{CO} = F_{CO-M} + F_{CO-C}},$$

where $\boldsymbol{F_{CO-M}}$ and $\boldsymbol{F_{CO-C}}$ are the additional forces provided by the surface tension of the cell membrane and the elastic force provided by the cytoplasm, respectively. The elastic force provided by the cell membrane was ignored due to its thickness being much less than the size of the cell.

Increasing the cross-sectional radius of the probe from $\boldsymbol{r}$ to $\boldsymbol{r + dr}$ yields the equations

$$\boldsymbol{dF_{CO-M} = 2\pi(r + dr)\gamma cos\theta - 2\pi r\gamma cos\theta = 2\pi\gamma cos\theta dr}$$

and

$$\boldsymbol{F_{CO-M} = \int_0^a 2\pi\gamma cos\theta dr = 2\pi\gamma cos\theta a = 2\pi\gamma cos\theta \frac{2}{\pi}\delta tan\theta = 4\gamma\delta sin\theta}.$$

For a conical probe,

$$\boldsymbol{F_{CO-C}(t) = \frac{2tan\theta\delta^2 E_C}{\pi(1-v)}\left(1 + \frac{\tau_\sigma - \tau_\varepsilon}{\tau_\varepsilon} e^{-\frac{t}{\tau_\varepsilon}}\right)}.$$

Thus,

$$\boldsymbol{F_{CO}(t) = F_{CO-M} + F_{CO-C} = 4\gamma\delta sin\theta + \frac{2tan\theta\delta^2 E_C}{\pi(1-v)}(1 + \frac{\tau_\sigma - \tau_\varepsilon}{\tau_\varepsilon} e^{-\frac{t}{\tau_\varepsilon}})}$$

and

$$\boldsymbol{F_{CO}(t) - F_{CO}(\infty) = \frac{2tan\theta\delta^2 E_C}{\pi(1-v)} \frac{\tau_\sigma - \tau_\varepsilon}{\tau_\varepsilon} e^{-\frac{t}{\tau_\varepsilon}}}.$$

Here, $\boldsymbol{\delta}$ can be calculated by the force-distance curve at approaching phase, so by fitting the equation $\boldsymbol{F_{CO}(t) - F_{CO}(\infty)}$ to the data, we were able to calculate the elastic modulus of the cytoskeleton network $\boldsymbol{E_C}$ and the relaxation times $\boldsymbol{\tau_\sigma}$ and $\boldsymbol{\tau_\varepsilon}$. And the apparent viscosity of cytosol was calculated by $\boldsymbol{\eta = E_C(\tau_\sigma - \tau_\varepsilon)}$.

And the membrane tension was calculated using the equation

$$\gamma = \frac{F_{CO}(\infty) - \frac{2tan\theta\delta^2 E_C}{\pi(1-v)}}{4\delta sin\theta}.$$

We also derived the formula

$$\frac{(F_{CO}(0) - F_{CO-C}(0))}{F_{CO-C}(0)} = sin\theta\pi(1-v)\frac{2\gamma}{E_C tan\theta\delta},$$

which also corresponds to finite element fitting results in Ding et al (Ding et al., 2018).

For instantaneous modulus,

$$E_I = \frac{2\gamma cos\theta\pi(1-v)}{\delta E_C} + E_C\frac{\tau_\sigma}{\tau_\varepsilon},$$

with this expression showing a decrease in instantaneous modulus with increasing indentation depth.

2. Supplementary Results

Considering that different culture medium may affect the mechanical properties of cells, some additional experiments were implemented. After all the experiments involved in the manuscript were completed, the five types of cells involved in this paper were re-cultured with 1640 (RPMI-1640 medium containing 10% FBS with 1% P/S and 0.5% L-glutamine) and DMEM (DMEM containing 10% FBS with 1% P/S and 0.5% glutamine) and characterized by AFM (among them, MCF-10A was additionally cultured with DMEM/F12 medium (DMEM/F12 plus 5% horse serum (HS) supplemented with 20 ng/mL epidermal growth factor, 0.5 μg/mL hydrocortisone, 10 μg/mL insulin, 1% non-essential amino acids and 1% P/S)). According to the analysis of the AFM characterization results, as is shown in **Supplementary Figure 2**, it was found that, except for MCF-10A cells (for MCF-10A cells cultured with 1640, DMEM and DMEM/F12, instantaneous modulus are 902.84±371.81 Pa, 900.94±333.75 Pa and 654.79±220.23 Pa respectively, relaxed modulus are 465.28±355.36 Pa, 482.26±275.39 Pa and 405.98±202.57 Pa respectively, cytoskeleton modulus are 26.81±18.56 Pa, 29.17±15.73 Pa and 8.11±5.26 Pa respectively, apparent viscosity are 94.03±51.10 Pa·s, 87.44±37.17 Pa·s and 39.34±17.64 Pa·s respectively and membrane tension are 0.32±0.20 mNm^{-1}, 0.32±0.15 mNm^{-1} and 0.30±0.15 mNm^{-1} respectively.) and MCF-7 cells (for MCF-7 cells cultured with 1640 and DMEM, instantaneous modulus are 348.04±107.91 Pa and 404.00±121.47 Pa respectively ($p=0.064$), relaxed modulus are 166.51±75.71 Pa and 256.88±98.77 Pa respectively ($p<0.001$), cytoskeleton modulus are 7.57±3.12 Pa and 6.00±3.73 Pa respectively ($p=0.082$), apparent viscosity are 35.06±10.92 Pa·s and 34.32±13.63 Pa·s respectively ($p=0.816$) and membrane tension are 0.12±0.05 mNm^{-1} and 0.19±0.06 mNm^{-1} respectively ($p<0.001$).), the mechanical properties of the remaining three cell types (for L929 cells cultured with 1640 and DMEM, instantaneous modulus are 523.85±96.35 Pa and 497.24±123.03 Pa respectively ($p=0.412$), relaxed modulus are 363.86±55.80 Pa and 330.76±96.30 Pa respectively ($p=0.217$), cytoskeleton modulus are 4.36±3.57 Pa and 5.23±3.43 Pa respectively ($p=0.381$), apparent viscosity are 26.03±6.62 Pa·s and 28.61±13.07 Pa·s respectively ($p=0.633$) and membrane tension are 0.24±0.05 mNm^{-1} and 0.24±0.06 mNm^{-1} respectively ($p=0.904$). For HC11 cells cultured with 1640 and DMEM, instantaneous modulus are 618.55±356.05 Pa and 666.61±363.59 Pa respectively ($p=0.607$), relaxed

modulus are 417.60±292.61 Pa and 396.08±224.51 Pa respectively (p=0.750), cytoskeleton modulus are 6.99±5.06 Pa and 6.70±4.02 Pa respectively (p=0.804), apparent viscosity are 31.19±16.40 Pa·s and 34.74±16.60 Pa·s respectively (p=0.409) and membrane tension are 0.25±0.06 mNm^{-1} and 0.27±0.13 mNm^{-1} respectively (p=0.442). For 4T1 cells cultured with 1640 and DMEM, instantaneous modulus are 350.95±184.30 Pa and 353.42±222.14 Pa respectively (p=0.963), relaxed modulus are 227.19±117.75 Pa and 229.05±165.41 Pa respectively (p=0.960), cytoskeleton modulus are 4.36±3.01 Pa and 4.06±3.05 Pa respectively (p=0.702), apparent viscosity are 22.37±16.20 Pa·s and 22.39±17.42 Pa·s respectively (p=0.996) and membrane tension are 0.16±0.07 mNm^{-1} and 0.14±0.08 mNm^{-1} respectively (p=0.422).) were not significantly different after being cultured in different media. For MCF-10A cells, there is no significant difference between the cells cultured with 1640 and DMEM (p=0.983 for instantaneous modulus, p=0.837 for relaxed modulus, p=0.697 for cytoskeleton modulus, p=0.570 for apparent viscosity and p=0.929 for membrane tension), but there is a significant difference between the cells cultured with 1640 and DMEM/F12 (p=0.003 for instantaneous modulus, p=0.432 for relaxed modulus, p<0.001 for cytoskeleton modulus, p<0.001 for apparent viscosity and p=0.662 for membrane tension) and the cells cultured with DMEM and DEME/F12 (p=0.001 for instantaneous modulus, p=0.229 for relaxed modulus, p<0.001 for cytoskeleton modulus, p<0.001 for apparent viscosity and p=0.677 for membrane tension). And as shown in **Supplementary Table 1,** there are significant differences between MCF-10A and MCF-7 cultured with 1640 and DMEM. Compared with MCF-7 cells with 1640 and DMEM medium, MCF-10A cells have higher instantaneous modulus, higher relaxation modulus, higher cytoskeleton modulus, higher apparent viscosity and higher membrane tension.

3. Supplementary Figures

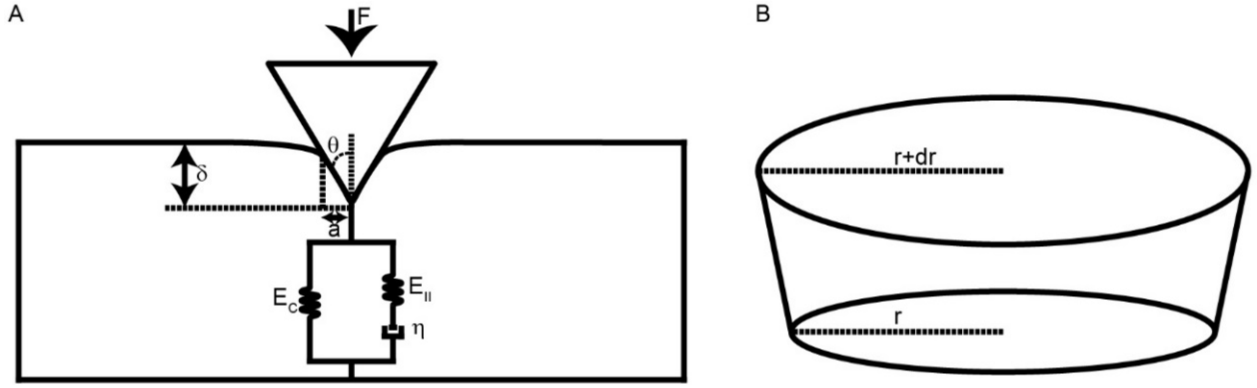

Supplementary Figure 1. (A) A physical model of the cell membrane, cytoskeleton and cytosol considered together for a conical probe. (B) The structure diagram of the probe when the cross section radius of the probe increases from $\boldsymbol{r}$ to $\boldsymbol{r+dr}$.

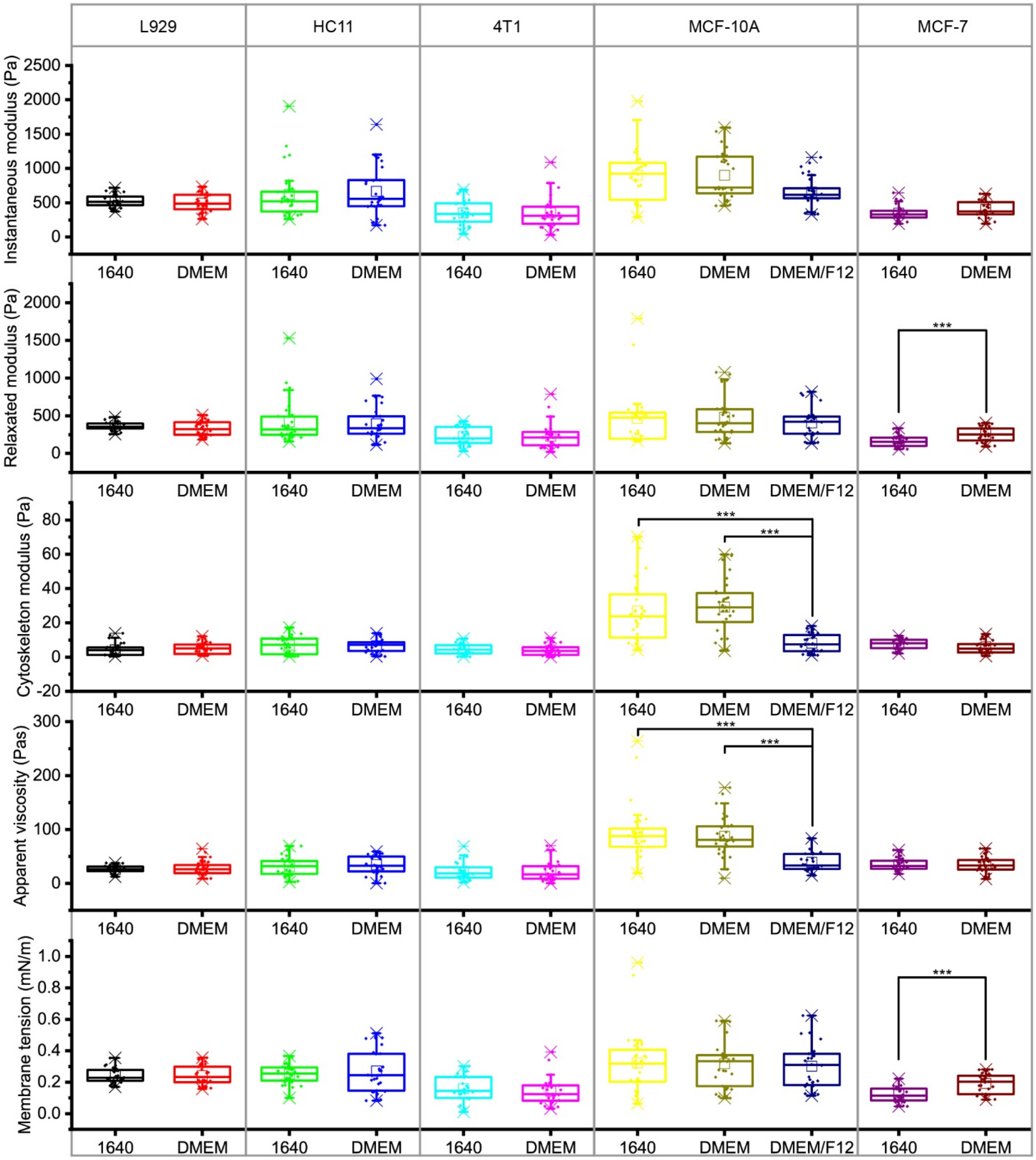

Supplementary Figure 2. Box plots of various parameters of various cells cultured in different medium. 1640, DMEM and DMEM/F12 represent cells cultured with 1640, DMEM and DMEM/F12 medium, respectively.

4. Supplementary Table

Supplementary Table 1. The statistical results of various parameters of MCF-10 and MCF-7 cells cultured in different media.

	E_I	E_R	E_C	η	γ
MCF-10A-1640 & MCF-7-1640	p<0.001	p<0.001	p<0.001	p<0.001	p<0.001
MCF-10A-1640 & MCF-7-DMEM	p<0.001	p=0.004	p<0.001	p<0.001	p=0.002
MCF-10A-DMEM & MCF-7-1640	p<0.001	p<0.001	p<0.001	p<0.001	p<0.001
MCF-10A-DMEM & MCF-7-DMEM	p<0.001	p<0.001	p<0.001	p<0.001	p<0.001

Ding, Y., Wang, J., Xu, G.-K., and Wang, G.-F. (2018). Are elastic moduli of biological cells depth dependent or not? Another explanation using a contact mechanics model with surface tension. *Soft Matter* 14(36), 7534-7541. doi: 10.1039/c8sm01216d.
